# Hematobiochemical, serological, and molecular detection of *Anaplasma marginale* in dromedary camels (*Camelus dromedarius*) in Al-Najaf desert, Iraq

**DOI:** 10.14202/vetworld.2023.1340-1345

**Published:** 2023-06-14

**Authors:** Ali Hussein Aldujaily, Nadia Abdul Hadee Abdul Ameer, Shatha Atta Abeed

**Affiliations:** 1 Department of Veterinary Clinical Sciences, Faculty of Veterinary Medicine, University of Kufa, Kufa, Al-Najaf, Iraq; 2 Department of Pharmacy, Al-Furat Al-Awsat Technical University, Kufa Technical Institute, Kufa, Al-Najaf, Iraq; 3 Department of Animal Production, Al-Furat Al-Awsat Technical University, Kufa Technical Institute, Kufa, Al-Najaf, Iraq

**Keywords:** *Anaplasma marginale*, biochemical parameters, dromedary Camel, hematological parameters, indirect enzyme-linked immunosorbent assay, polymerase chain reaction

## Abstract

**Background and Aim:**

Anaplasmosis, an underestimated disease transmitted by ticks, is widespread in ruminants, such as the Arabian camel (dromedary camel). This study aimed to examine the presence of *Anaplasma marginale* in dromedary camels in the Al-Hiadyia region of the Al-Najaf desert, Iraq, using serological and molecular tests. Moreover, hematological and biochemical changes in infected animals were compared with those in healthy controls.

**Materials and Methods:**

The study was conducted on 30 healthy and 260 infected camels with severe anemia, pale mucus membranes, and progressive emaciation to investigate antibodies against *A. marginale* using indirect enzyme-linked immunosorbent assay, followed by polymerase chain reaction for selected positive samples targeting a specific region of *A. marginale* major surface protein 5 (MSP5). In addition, hematological and biochemical parameters were measured to indicate the effect of the disease on blood profile, mineral status, and liver and kidney functions.

**Results:**

Enzyme-linked immunosorbent assay analysis and microscopic examination revealed that 115/260 (44.23%) and 87 (33.46%) camels were positive for *Anaplasma* spp., respectively. The MSP5 gene, which is unique to *A. marginale*, was amplified. The results of hematological analysis indicated a significant decrease in total red blood cells, hemoglobin, and packed cell volume and a significant increase in mean corpuscular volume in infected camels, but no difference in mean corpuscular hemoglobin concentration. Moreover, there was a significant increase in total white blood cells count, lymphocytes, erythrocyte sedimentation rate, and platelets. The results of biochemical analysis indicated a significant increase in the levels of aspartate aminotransferase, alanine aminotransferase, and alkaline phosphatase, blood urea nitrogen, creatinine, and iron and a decrease in copper in infected camels. Cholesterol and triglyceride showed no significant variations between healthy and diseased camels.

**Conclusion:**

To the best of our knowledge, this is the first molecular study to demonstrate the presence of *A. marginale* in dromedary camels in Iraq. The MSP5 gene is a valuable and unique diagnostic target for identifying *A. marginale*.

## Introduction

The dromedary camel (*Camelus dromedarius*), also known as the one-humped camel, is found in several places globally. The camel is an adaptable animal found in semi-arid and desert regions [1]. Anaplasmosis is one of the most prevalent tick-borne, hemoparasitic diseases of domestic animals and ruminants in tropical and subtropical regions worldwide, and is caused by intra-erythrocytic rickettsial pathogens of the genus *Anaplasma* (Order: Rickettsial; Family: Anaplasmataceae) [2].

Anaplasmosis is characterized by fever, progressive hemolytic anemia produced by erythrocyte invasion and extravascular hemolysis, gastrointestinal issues, weight loss, emaciation, and abortion and causes great economic loss. Intracellular infections caused by *Anaplasma* spp. promote hemolysis outside circulation. Intracellular *Anaplasma* species include *Anaplasma marginale, Anaplasma centrale, Anaplasma ovis, Anaplasma capra*, and *Candidatus Anaplasma camelii*. *Anaplasma marginale* causes anaplasmosis in cattle and infects dromedaries and cervids [3].

However, *A. marginale* is the most prevalent virulent agent in mild tropical and subtropical areas and causes acute anaplasmosis, characterized by progressive hemolytic anemia, high morbidity and mortality rates, and considerable economic loss [4]. Although *A*. *centrale* can cause moderate anemia, clinical outbreaks in the field are exceedingly rare; as a result, it is exploited as a live vaccine in tropical and subtropical settings to protect against the pathogenic *A. marginale* [5].

The transmission of *Anaplasma* can occur either mechanically, such as through fly bites, or biologically, such as through >20 species of blood-sucking ticks [6]. Animals recovering from acute anaplasmosis become chronically infected with *A. marginale* and serve as a reservoir within the herd, because they are infected for life [7].

Conventionally, thin blood smears stained with Giemsa are used to diagnose anaplasmosis in animals suspected of having acute disease; however, these smears are not suitable for detecting subclinical infection in carriers [8]. Alternatively, serological assays, including the card agglutination test, complement fixation test, indirect fluorescent antibody test, and enzyme-linked immunosorbent assay (ELISA), can be used to detect *Anaplasma*-specific antibodies [9]. Enzyme-linked immunosorbent assay has been used in a variety of ruminants, including cattle, buffalo, sheep, and camels, for diagnosing infections caused by *A. marginale* [10].

These diagnostic approaches have some limitations, including limited sensitivity and inability to differentiate between *Anaplasma* species. Polymerase chain reaction (PCR) is more sensitive and specific at the species level for identifying *Anaplasma* DNA and may be used in phylogenetic research [11].

An additional important aspect of the diagnostic process for anaplasmosis, in particular for *A. marginale*, must be considered. Animals that make it through the first stage of the disease are considered carriers, because they have persistent infection and seldom display clinical signs [12]. In carriers, a diagnosis based on Giemsa-stained blood smears is not sufficient; however, a diagnosis based on serological and molecular-based approaches is more accurate [9].

Despite the rising interest in camel anaplasmosis by the scientific community, few studies have been conducted in Iraq to identify the species and other variables responsible for the disease. This study aimed to examine the presence of *A. marginale* in dromedary camels in the Al-Hiadyia region of the Al-Najaf desert using serological and molecular testing. In addition, hematological and biochemical alterations in infected animals were compared with those of healthy controls.

## Materials and Methods

### Ethical approval and Informed consent

A Local Ethics Committee examined and approved (Approval no. 21735 dated 13/1/2021) the study protocol, subject information, and informed consent form (Faculty of Veterinary Medicine University of Kufa). All participants in this study were informed and their oral consent was acquired for conducting tests and publishing the results.

### Study period and location

The study was conducted from September 2021 to April 2022 in the Al-Najaf province, Iraq.

### Hematological and biochemical analyses

A total of 260 camels aged between 4 and 11 years who had severe anemia and jaundice, as well as 30 camels that were clinically normal and served as controls. Ten ml of jugular venous blood was collected from each study camel. The blood samples were sent to the clinical pathology laboratory for hematological and biochemical analyses. The hematological parameters of whole blood, including red blood cells (RBCs), white blood cells (WBCs), hemoglobin (Hb), packed cell volume (PCV), mean corpuscular volume (MCV), mean corpuscular hemoglobin concentration (MCHC), and platelets (Plts), were analyzed using the fully-automated Vetscan HM5 Hematology Analyzer (ABAXIS Company, Union City, California USA). Erythrocyte sedimentation rate (ESR) was measured using Westergren tubes [13].

Blood samples in plain tubes were centrifuged at (1006× *g* for 15 min. All samples were frozen at −20°C and sent to the Laboratory of Kufa, Faculty of Veterinary Medicine, for analysis. Levels of aspartate aminotransferase (AST), alanine aminotransferase (ALT), and alkaline phosphatase (ALP) were measured through biochemical tests. Cholesterol, triglyceride, creatinine, and blood urea nitrogen (BUN) levels were evaluated by ultraviolet spectrophotometry [14]. Iron and copper were quantified using atomic absorption spectrophotometry (Auto analyserbt 35i RingeLsan Co. Gaziemir-Izmir-Turkey).

### Parasitological examination

Thin blood smears were prepared from 260 camels, stained with Giemsa, and examined under a light microscope (SN/OF1807, Olympus, Japan) to determine the presence of intra-erythrocytic *Anaplasma* inclusion bodies [15].

### Serological test

A total of 260 camel serum samples were collected. An indirect ELISA test was performed to identify *A. marginale* antibodies using an *A. marginale*-Ab ELISA Kit (Svanova Biotech AB, Sweden). The results were evaluated at 405 nm using a Clindiag MR-96 microplate reader.

### DNA extraction

gSYNC DNA Extraction Kit (Geneaid, USA) was used to extract genomic DNA from whole blood samples positive for *Anaplasma* inclusion bodies by microscopy examination and ELISA, according to the manufacturer’s instructions, and stored at −20°C until use.

### Molecular detection of major surface protein 5 (MSP5) in *A. marginale*

*Anaplasma marginale* MSP5 gene was amplified using extracted DNA and the indicated primer sets AMF: 5′-ACAGGCGAAGAAGCAGACAT-3′ and AMR: 5′-ATAAATGGGAACACGGTGGA-3′ [8, 16].

The PCR amplifications were performed in an Eppendorf Master cycler thermocycler with a total reaction volume of 25 μL. The reaction contained 1 μL of each primer, 2.5 μL of Fast Start DNA Master (GoTaq Green Master Mix, Promega, USA), 1 μL MgCl_2_, 5 μL of template DNA, and 14.5 μL of DNase-free water.

The following approach was used to amplify the MSP5 gene: Initial denaturation at 98°C for 5 min; followed by 35 cycles of denaturation at 98°C for 2 s, annealing at 53°C for 30 s, and extension at 72°C for 30 s; and a final extension at 72°C for 1 min. The PCR products were placed onto a 2% agarose gel and stained with 0.5 μg/mL ethidium bromide. The amplified DNA was electrophoresed at 100 V for 60 min on horizontal electrophoresis equipment. The gels were photographed using the gel documentation method (Biometra, Germany). A 100-bp DNA ladder was used to estimate fragment size.

### Statistical analysis

Data were investigated using the Statistical Package for the Social Sciences version 26 (IBM Corp., NY, USA). The data were subjected to statistical analysis of variance using independent samples t-test and Chi-square test. The data were expressed as mean ± standard error (SE); p < 0.05 was considered statistically significant. The least significant difference, also known as the minimal significant difference test, was used to identify differences between the groups.

## Results

**Figure-1:**
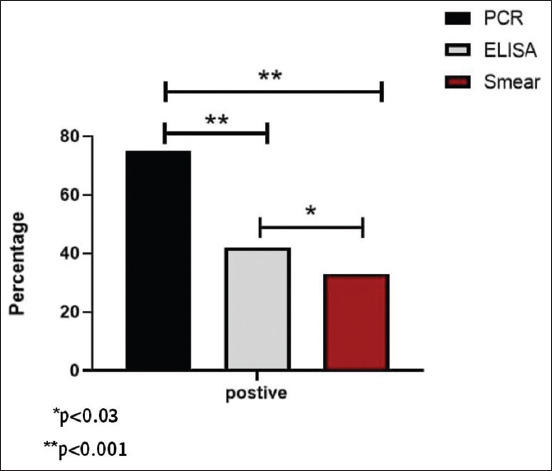
Comparison of the enzyme-linked immunosorbent assay, microscopic examination, and polymerase chain reaction tests.

**Figure-2:**
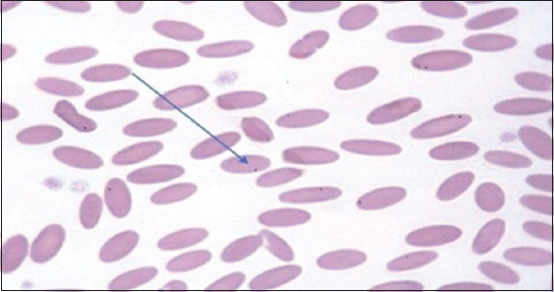
Giemsa-stained micrograph of camel blood smear.

Enzyme-linked immunosorbent assay analysis and microscopic examination revealed that 115/260 (44.23%) and 87/256 (33.46%) samples were positive, respectively (Figure-1). Reddish-violet, pleomorphic, and dot-like forms of *Anaplasma* spp. were detected in infected erythrocytes, with varying sizes from 0.2 to 0.4 μm (Figure-2).

**Figure-3:**
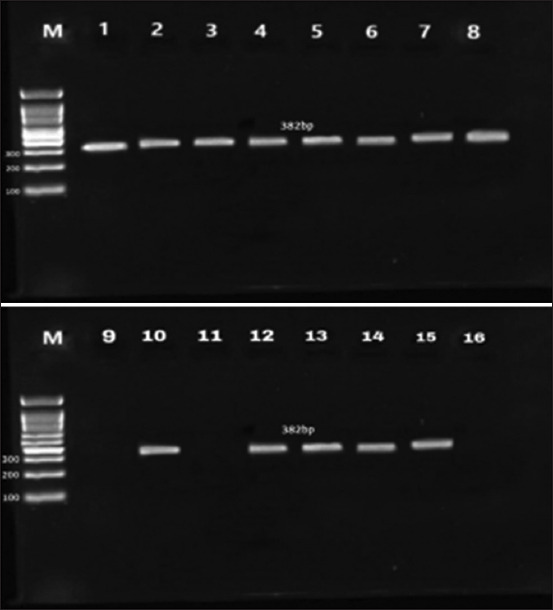
The effective amplification of a specific major surface protein 5 *Anaplasma marginale* gene by electrophoresis of polymerase chain reaction product on a 2% agarose gel stained with ethidium bromide. Lanes M: 100–1000 bp molecular weight marker; Lanes 1–8, 10, 12–15 positive band at 382 bp for *A. marginale* camel isolates; lanes 9, 11 and 16: negative band (no DNA).

DNA analysis of the 87 cases that tested positive with ELISA and microscopic examination revealed 65 (74.71%) positive cases (Figure-1). Isolates used for genetic analysis contained a 382 bp band unique to the MSP5 gene of *A. marginale* (Figure-3).

The range and mean ± SE of hematological parameters in clinically normal camels and *Anaplasma*-infected camels are shown in Table-1. Compared with healthy camels, *A. marginale*-infected camels exhibited a significant decrease in total RBC count, Hb, and PCV; a significant increase in MCV, ESR, and Plts; and no difference in MCHC. In addition, there was a significant increase in total WBC count and lymphocytes and a significant decrease in neutrophils, but no significant difference in monocytes and eosinophils was oberved between infected and healthy camels.

**Table-1:** The hematological parameters for normal and *Anaplasma marginale*-infected camels; means ± standard error.

Parameters	Groups		Significane (2-tailed)

	Normal camels (n = 30)	Infected camels (n = 115)	
Red blood cells×10 µL	10554 ± 103.80*	7189 ± 100.5	0.000
Hemoglobin (g/dL)	11.9 ± 0.07*	6.6 ± 0.08	0.000
Packed cell volume (%)	30.3 ± 0. 1*	21.2 ± 0.2	0.000
Mean corpuscular volume (fL)	28.6 ± 0.21	32.9 ± 0.55*	0.000
Mean corpuscular hemoglobin concentration (g/dL)	39.5 ± 0.1	39.3 ± 0.2	0.000
Erythrocyte sedimentation rate mm/8 h	8.1 ± 0.2	15.4 ± 0.5*	0.000
Platelets×10^3^µL	174.5 ± 6.4	291.6 ± 6.1*	0.000
White blood cells (10^3^/µL)	10360 ± 90.3	12399 ± 223.8*	0.001
Neutrophils	42.4 ± 0.40*	31.6 ± 0.49	0.000
Lymphocytes	44.9 ± 0.55	52.9 ± 0.66*	0.000
Monocytes	8.1 ± 0.30	7.6 ± 0.34	0.145
Eosinophils	5.2 ± 0.27	4.9 ± 0.28	0.359

The ranges and means ± SE of biochemical parameters revealed a significant increase in ALT, AST, ALP, BUN, creatinine, iron, and a significant decrease in copper. In contrast, no significant difference was observed in cholesterol and triglyceride between camels infected with *A. marginale* and healthy camels (Table-2).

**Table-2:** The biochemical parameters of normal and *Anaplasma marginale*-infected camels; mean ± standard error.

Parameters	Groups		Significance (2-tailed)

	Normal camels (No. 30)	Infected camels (No. 115)	
Alanine aminotransferase U/L	44.89 ± 2.63	70.89 ± 3.23*	0.001
Aspartate aminotransferase U/L	9.49 ± 0.37	18.97 ± 0.54*	0.002
Alkaline phosphatase U/L	68.90 ± 4.57	96.79 ± 3.88*	0.001
Cholesterol (mg/dL)	22.40 ± 0.99	21.70 ± 1.01	0.102
Triglyceride (mg/dL)	34.00 ± 1.57	32.92 ± 1.67	0.108
Blood urea nitrogen (mg/dL)	30.29 ± 1.23	49.58 ± 1.89*	0.003
Creatinine (mg/dL)	1.96 ± 0.05	2.65 ± 0.06	0.101
Iron (µmol/L)	13.1 ± 0.39	18.1 ± 0.74*	0.002
Copper (µmol/L)	11.01 ± 0.38*	6.98 ± 0.34	0.000

## Discussion

Anaplasmosis is a tick-borne, hemolytic disease caused by Gram-negative, obligate intracellular, shell-shaped bacteria, 0.4–0.5 μm in size, affecting ruminants worldwide, including the Middle East [17]. The majority of *A. marginale* infections in camels have been detected by the use of traditional blood testing using stained blood smears. In contrast, other *Anaplasma* species in camels have been detected using serological or molecular approaches [18].

The total detection rate of *Anaplasma* in this study was higher than that reported by others from Arabian camels in Saudi Arabia (40.55% and 42.39%, respectively) [8, 19], as well as Egypt (34.1%) [11] and Morocco (39.62%) [20].

The analysis of Giemsa-stained blood smears was less sensitive than PCR-based detection techniques. This might be attributed to sampling subclinical or chronically infected animals, which frequently had low quantities of infected erythrocytes. Another explanation could be the method’s reliance on microscopic imaging of the *A. marginale* intra-erythrocytic stage [21]. Enzyme-linked immunosorbent assay showed good sensitivity but low specificity compared with PCR. Enzyme-linked immunosorbent assay depends on the health of the animal and the ability of its immune system to produce antibodies against this changeable antigenic pathogen.

The first investigation to report and confirm the presence of *A. marginale* in dromedaries using molecular methods was conducted by Azmat *et al*. [22]. Major surface proteins are highly conserved in Anaplasmataceae. *Anaplasma marginale* MSP5 can be used to identify and construct phylogenetic trees [16], as well as to identify carriers [11].

The differences in the prevalence or detection rates of anaplasmosis reported could be due to several factors, including sample size, collection methodology, examination method, and a number of vector ticks that live in the area where the research was conducted, as well as conditions of environment and animal management. Blood samples for this study were collected by veterinarians who suspected “blood parasite infection,” which may explain the high detection rate.

To characterize the species responsible for anaplasmosis in Al-Najaf governorate, Iraq, and to explain the condition of the illness, further sequencing of PCR products acquired in this study is required. This can be accomplished using samples from various ruminant species in addition to ticks and other gene targets.

The mean values of hematological parameters in clinically healthy camels were found to lie within the normal range, as reported by Brooks *et al*. [23]. Consequently, we used a control group from the same herds. These animals were raised under comparable food systems, management, and environmental circumstances throughout the study.

However, *A. marginale*-infected camels exhibited a significant reduction in total RBC, Hb, and PCV, along with an increase in MCV and no significant change in MCHC. These data suggest hemolytic anemia and are comparable with that documented by Ismael *et al*. [19] and Khalafalla and Hussein [24].

The pathogenesis of anaplasmosis depends on the infection of mature erythrocytes. Anaplasmosis leads to an increase in morphological variations and osmotic fragility of RBCs, which makes them susceptible to phagocytosis by reticuloendothelial cells, resulting in mild-to-severe hemolytic anemia and icterus without hemoglobinemia and hemoglobinuria [6]. Hemolytic anemia results from increased reticulocyte destruction and is typically regenerative [25, 26].

Values of MCV and MCHC from infected animals indicated macrocytic normochromic anemia, which demonstrates that the hemopoietic system was activated in response to erythrophagocytosis, consistent with the findings of Johns and Heller [26] and Valli *et al*. [27].

The ESR of normal camels after 8 h was in accordance with that reported by Faye and Bengoumi [28]. Although the substantial increase in ESR values in camels with anemia may result from the correlation between RBC sedimentation and the severity of anemia, it is also attributable to a decrease in PCV [29].

The platelet counts observed in this study in normal camels were consistent with the findings recorded by Tornquist [30]. Although a considerable rise in Plts was seen in infected camels, this may be attributed to anemia [31].

Furthermore, the outcome of normal total WBCs, lymphocytes, neutrophils, monocytes, and eosinophils revealed a non-significant difference from the values recorded by Weiss and Wardrop [32]. Leukocytosis and lymphocytosis in infected camels might be related to the activation of lymphoid tissues and stem cells in the bone marrow due to *Anaplasma* infection and toxins released by *Anaplasma* [33].

In contrast, the mean values of biochemical parameters in clinically healthy camels were within normal ranges, as reported by Weiss and Wardrop [32] and Al-Dhalimy *et al*. [34]. Increased AST, ALT, and ALP levels in infected camels compared with healthy camels may indicate hepatic dysfunction.

In addition, significant hemolysis may occur, which when coupled with hypoxia, may result in glomerular dysfunction and hepatic cell degeneration, thereby increasing the levels of AST and ALT. These findings are consistent with Khalafalla and Hussein [24] and Abbas *et al*. [31].

The increased BUN level may suggest indirect injury to renal tissue and the presence of globin catabolizes released from Hb lysis by the reticuloendothelial system through erythrophagocytosis [35]. Furthermore, increased serum creatinine levels may be due to kidney dysfunction and muscle catabolism [36]. The non-significant changes in cholesterol and triglyceride concentrations observed in this study were consistent with those reported by Wellman and Radin [37].

Normal serum copper and iron values obtained in this study are close to the normal limit obtained by Abdelrahman *et al*. [38]. The serum iron and copper levels can reveal the status of mineral nutrients and their coordinated antioxidant role with antioxidant enzyme activities [39].

In this study, increased serum iron in infected camels was attributable to hemolytic anemia. Abnormal RBCs are identified by phagocytosed macrophages in the bone marrow, spleen, and liver. Macrophages break down Hb into globin and heme, hence elevating blood iron levels [40]. The decrease in serum copper found in this study is consistent with the findings of Esmaeilnejad *et al*. [39]. According to Ismael *et al*. [19], *A. marginale* is the only *Anaplasma* species that causes disease in dromedaries, which is consistent with our findings.

## Conclusion

Anaplasmosis has a negative impact on the health of camels and affects their hematological and biochemical parameters. Positive tests for the presence of *Anaplasma* species were conducted on dromedary camels in Al-Hiadyia, Al-Najaf, Iraq. Amplification of the particular MSP5 gene proved that the in-issue organism belonged to the species *A. marginale*.

## Authors’ Contributions

AHA: Collected the samples and data. NAHAA and SAA: Data analysis. AHA, NAHAA, and SAA: Laboratory tests and drafted and revised the manuscript. All authors have read, reviewed, and approved the final manuscript.

## References

[1] Ali A, Baby B, Vijayan R (2019). From desert to medicine:A review of camel genomics and therapeutic products. Front. Genet.

[2] Mubashir M, Tariq M, Khan M.S, Safdar M, Özaslan M, Imran M, Junejo Y (2022). Review on anaplasmosis in different ruminants. Zeugma Biol. Sci.

[3] Rar V, Tkachev S, Tikunova N (2021). Genetic diversity of *Anaplasma* bacteria:Twenty years later. Infect. Genet. Evol.

[4] Ashraf S, Parveen A, Awais M.M, Gillani Q, Aktas M, Ozubek S, Iqbal F (2021). A report on molecular detection and phylogenetic evaluation of *Anaplasma marginale* in ticks and blood samples collected from cattle in district Layyah in Punjab (Pakistan). Curr. Microbiol.

[5] Hove P, Khumalo Z.T.H, Chaisi M.E, Oosthuizen M.C, Brayton K.A, Collins N.E (2018). Detection and characterisation of *Anaplasma marginale* and *A. centrale* in South Africa. Vet. Sci.

[6] Constable P.D, Hinchcliff K.W, Done S.H, Grünberg W (2017). Veterinary Medicine.

[7] Dahmani M, Davoust B, Sambou M, Bassene H, Scandola P, Ameur T, Mediannikov O (2019). Molecular investigation and phylogeny of species of the Anaplasmataceae infecting animals and ticks in Senegal. Parasit. Vectors.

[8] Omer E, Elfehid M, Alwazan A, Alouffi A.S, Alshammari F.A, Eldesoukey I, Sultan K (2022). Molecular detection of *Anaplasma marginale* in Arabian camels (*Camelus dromedarius*) in Riyadh, Saudi Arabia. J. Saudi Soc. Agric. Sci.

[9] Bisen S, Aftab A, Jeeva K, Silamparasan M, Yadav S, Chandra D, Raina O.K (2021). Molecular and serological detection of *Anaplasma* infection in carrier cattle in north India. Vet. Parasitol. Reg. Stud. Rep.

[10] Al-Ethafa L.F, Al-Galebi A.A, Al-Hassani M.K (2019). Microscopic-serologic survey of *Anaplasma marginale* rickettsia in buffaloes in Al-Qadisiyah and Babylon governorates, Iraq. J. Pure Appl. Microbiol.

[11] Parvizi O, El-Adawy H, Roesler U, Neubauer H, Mertens-Scholz K (2020). Performance analysis of *Anaplasma* antibody competitive ELISA using the ROC curve for screening of anaplasmosis in camel populations in Egypt. Pathogens.

[12] Kocan K.M, de la Fuente J, Blouin E.F, Coetzee J.F, Ewing S.A (2010). The natural history of *Anaplasma marginale*. Vet. Parasitol.

[13] Aldujaily A.H, Abeed S.A, Ameer N.A.H.A (2020). Diagnosis of polioencephalomalacia in dromedary camels (*Camelus dromedarius*) from Al-Hiadyia. Plant Arch.

[14] Kerr M.G (2008). Veterinary Laboratory Medicine:Clinical Biochemistry and Hematology.

[15] Sharma A, Singla L.D, Kaur P, Bal M.S, Batth B.K, Juyal P.D (2013). Prevalence and haemato-biochemical profile of *Anaplasma marginale* infection in dairy animals of Punjab (India). Asian Pac. J. Trop. Med.

[16] Ganguly A, Maharana B.R, Ganguly I, Kumar A, Potliya S, Arora D, Bisla R.S (2018). Molecular diagnosis and haemato-biochemical changes in *Anaplasma marginale*-infected dairy cattle. Indian J. Anim. Sci.

[17] Soosaraei M, Haghi M.M, Etemadifar F, Fakhar M, Teshnizi S.H, Asfaram S, Esboei B.R (2020). Status of *Anaplasma* spp. infection in domestic ruminants from Iran:A systematic review with meta-analysis. Parasite. Epidemiol. Control.

[18] Alshahrani M.Y, Alanazi A.D, Alouffi A.S, Abdullah H.H.A.M, Allam A.M, Mahmoud M.S, Alkhathami A.G (2020). Molecular detection of *Candidatus*
*Anaplasma camelii* in camels (*Camelus dromedarius*) from Asir Province, Saudi Arabia. Trop. Biomed.

[19] Ismael A.B, Swelum A.A.A, Khalaf A.F, Alowaimer A.N (2016). First evidence of natural anaplasmosis in *Camelus dromedarius* in Saudi Arabia. J. Camel Pract. Res.

[20] Lbacha H.A, Zouagui Z, Alali S, Rhalem A, Petit E, Ducrotoy M.J, Maillard R (2017). “*Candidatus Anaplasma camelii*”in one-humped camels (*Camelus dromedarius*) in Morocco:A novel and emerging *Anaplasma* species?. Infect. Dis. Poverty.

[21] Silaghi C, Santos A.S, Gomes J, Christova I, Matei I.A, Walder G, Dumler J.S (2017). Guidelines for the direct detection of *Anaplasma* spp. in diagnosis and epidemiological studies. Vector Borne Zoonotic Dis.

[22] Azmat M, Ijaz M, Farooqi S.H, Ghaffar A, Ali A, Masud A, Zhang H (2018). Molecular epidemiology, associated risk factors, and phylogenetic analysis of anaplasmosis in camel. Microb. Pathog.

[23] Brooks M.B, Harr K.E, Seelig D.M, Wardrop K.J, Weiss D.J (2022). Schalm's Veterinary Hematology.

[24] Khalafalla A.I, Hussein M.F (2021). Infectious Diseases of Dromedary Camels.

[25] Stevens A, Lowe J.S, Scott I (2012). Veterinary Hematology. A Diagnostic Guide and Color Atlas.

[26] Johns J, Heller M (2021). Hematologic conditions of small ruminants. Vet. Clin. North Am. Food Anim. Pract.

[27] Valli V.E.O, Kiupel M, Bienzle D, Wood R.D (2015). Hematopoietic system. Jubb, Kennedy, and Palmer's Pathology of Domestic Animals.

[28] Faye B, Bengoumi M (2018). Camel Clinical Biochemistry and Hematology.

[29] Jain N.C (1986). Haematological techniques. Schalm's Veterinary Hematology.

[30] Tornquist S.J (2022). Hematology of camelids. Schalm's Veterinary Hematology.

[31] Abbas M.Z, Saqib M, Deeba F, Sajid M.S (2020). Impact of subclinical anaplasmosis on hemato-biochemical parameters of naturally infected camels (*Camelus dromedarius*). Pak. J. Agric. Sci.

[32] Weiss D.J, Wardrop K.J (2011). Lymphopoiesis, neutrophil structure and biochemistry. Schalm's Veterinary Hematology.

[33] Al-Obaidi Q.T, Hasan S.D, Alsaad K.M (2021). Clinical, haematological and blood biochemical parameters in Arabian one-humped camels (*Camelus dromedarius*) with *Babesia caballi* infection. Bulg. J. Vet. Med.

[34] Al-Dhalimy A.M.B, Aldhalemi A.A, Aldhalemi M.A, Bustani G.S (2020). Study of the deficiency of some elements and some vital variables in camel's blood. Plant Arch.

[35] Hajinejad-Bamroud G, Maghsoudi A, Rokouei M, Jahantigh M, Masoudi A. A (2020). Comparison of anatomical and blood biochemical parameters of Iranian racing and dual-purpose camels (*Camelus dromedarius*). Iran. J. Vet. Med.

[36] Nasreldin N, Ewida R.M, Hamdon H, Elnaker Y.F (2020). Molecular diagnosis and biochemical studies of tick-borne diseases (anaplasmosis and babesiosis) in Aberdeen Angus Cattle in New Valley, Egypt. Vet. World.

[37] Wellman M.L, Radin M.J (2022). Hematology, clinical biochemistry, and fluid analysis. Med. Surg. Camelids.

[38] Abdelrahman M.M, Alhidary I.A, Alobre M.M, Matar A.M, Alharthi A.S, Faye B, Aljumaah R.S (2022). Regional and seasonal variability of mineral patterns in some organs of slaughtered one-humped camels [*Camelus dromedarius*] from Saudi Arabia. Animals (Basel).

[39] Esmaeilnejad B, Tavassoli M, Samiei A, Hajipour N, Imani-Baran A, Farhang-Pajuh F (2018). Evaluation of oxidative stress and antioxidant status, serum trace mineral levels and cholinesterases activity in cattle infected with *Anaplasma marginale*. Microb. Pathog.

[40] Li W, Guo R, Song Y, Jiang Z (2022). Corrigendum:Erythroblastic Island macrophages shape normal erythropoiesis and drive associated disorders in erythroid hematopoietic diseases. Front. Cell Dev. Biol.

